# S2P2—the chloroplast-located intramembrane protease and its impact on the stoichiometry and functioning of the photosynthetic apparatus of *A. thaliana*

**DOI:** 10.3389/fpls.2024.1372318

**Published:** 2024-03-15

**Authors:** Maria Ciesielska, Małgorzata Adamiec, Robert Luciński

**Affiliations:** Department of Plant Physiology, Faculty of Biology, Institute of Experimental Biology, Adam Mickiewicz University in Poznań, Poznań, Poland

**Keywords:** *Arabidopsis thaliana*, chloroplast, S2P2, intramembrane protease, photosystem II, photoinhibition

## Abstract

S2P2 is a nuclear-encoded protease, potentially located in chloroplasts, which belongs to the zinc-containing, intramembrane, site-2 protease (S2P) family. In *A. thaliana* cells, most of the S2P proteases are located within the chloroplasts, where they play an important role in the development of chloroplasts, maintaining proper stoichiometric relations between polypeptides building photosynthetic complexes and influencing the sensitivity of plants to photoinhibitory conditions. Among the known chloroplast S2P proteases, S2P2 protease is one of the least known. Its exact location within the chloroplast is not known, nor is anything known about its possible physiological functions. Therefore, we decided to investigate an intra-chloroplast localization and the possible physiological role of S2P2. To study the intra-chloroplast localization of S2P2, we used specific anti-S2P2 antibodies and highly purified chloroplast fractions containing envelope, stroma, and thylakoid proteins. To study the physiological role of the protease, we used two lines of insertion mutants lacking the S2P2 protease protein. Here, we present results demonstrating the thylakoid localization of S2P2. Moreover, we present experimental evidence indicating that the lack of S2P2 in *A. thaliana* chloroplasts leads to a significant decrease in the level of photosystem I and photosystem II core proteins: PsaB, PsbA, PsbD, and PsbC, as well as polypeptides building both the main light-harvesting antenna (LHC II), Lhcb1 and Lhcb2, as well as Lhcb4 and Lhcb5 polypeptides, constituting elements of the minor, peripheral antenna system. These changes are associated with a decrease in the number of PS II–LHC II supercomplexes. The consequence of these disorders is a greater sensitivity of *s2p2* mutants to photoinhibition. The obtained results clearly indicate that the S2P2 protease is another thylakoid protein that plays an important role in the proper functioning of *A. thaliana* chloroplasts, especially in high-light-intensity conditions.

## Introduction

S2P2 is a nuclear-encoded protein, belonging to the family of intramembrane S2P proteases. These types of proteases occur ubiquitously in all living organisms (Kinch et al., 2006) and are believed to be involved in the regulation of gene expression by releasing, via proteolytical cleavage, membrane-anchored transcription factors (Lal and Caplan, 2011). The S2P family includes the highly hydrophobic intramembrane, zinc-dependent metalloproteases, which contain, within their structure, several transmembrane domains. Within these domains, two motifs crucial for proteolytic activity are present. The first of them is HExxH, which is responsible for binding the zinc ion. The second—LDG motif—also contributes to zinc binding and the proteolytic activity of S2P. Structural analyses indicate that the proper functioning of the LDG motif is largely dependent on the strongly conserved aspartic residue present in it (Adamiec et al., 2017; Danyukova et al., 2022).

The S2P protease was first isolated from the mammalian cells as an element essential for controlling lipid metabolism. The protease participated in the activation of the sterol regulatory element binding proteins (SREBPs), which are membrane-associated transcription factors (Brown and Goldstein, 1997).

In plants, the first identified and described S2P protease was EGY1 (ethylene-dependent gravitropism-deficient and yellow-green 1; Chen et al., 2005). The lack of this protease leads to pleiotropic effects in *A. thaliana* cells. Two of the most characteristic features of *egy1* mutants are the yellow-green pigmentation of rosette leaves and deficiencies in ethylene-dependent gravitropism, which results from the underdevelopment of chloroplast and lower starch content (Chen et al., 2005; Guo et al., 2008). From this phenotype, the protease derived its name EGY1 (ethylene-dependent gravitropism-deficient and yellow-green 1). The EGY1 protease was also found to be involved in response to ammonium and phosphate stresses (Li et al., 2012; Yu et al., 2016).

In addition to Egy1 in the *A. thaliana* genome, five other *S2P* genes were identified, namely, *Egy2*, *S2P2*, *AraSP*, *AT4G20310*, and *EGY3* genes (Adamiec et al., 2017). In the case of EGY1 and EGY2, there is clear experimental evidence of their proteolytic activity, despite large problems with identifying physiological substrates for these proteases. EGY3 is classified as the so-called pseudoprotease, due to the lack of the key motif for protease activity. However, despite the lack of proteolytic activity, it has been shown that EGY3 plays an important role in the stabilization of other chloroplast proteins, such as CAO (Adamiec et al., 2020). In turn, AraSP and S2P2 are considered potentially active (Bölter et al., 2006; Zybailov et al., 2008) due to the presence in their structure of all domains characteristic of S2P proteases, although experimental evidence for this activity has not been presented so far. Most of the S2P proteases are located in chloroplast membranes. The only known exception is the protein encoded by the *AT4G20310* gene which was found in the Golgi membrane. In turn, the intra-chloroplast localization of individual S2P proteins is diverse: Egy1 and Egy2 proteases were proven to be located in thylakoid membranes, whereas AraSP was found among proteins of the chloroplast envelope (Adamiec et al., 2017). Nevertheless, S2P2 remains a protein for which there is currently no information about its exact location within the chloroplast.

The physiological role of chloroplast S2P proteases and their mechanism of action remain poorly understood. It has been demonstrated that AraSP is crucial for plant development and chloroplast biogenesis. The mutants deprived of this protein displayed severe phenotyping abnormalities: small size, poorly developed roots, red cotyledons, lack of apical meristem, and a lifetime reduced to less than 20 days (Bölter et al., 2006). The molecular mechanism underlying this phenotype remains, however, unknown. The *egy2* mutants are characterized by shorter hypocotyls, lower fatty acid content, and increased sensitivity for photoinhibitory conditions (Chen et al., 2012; Adamiec et al., 2018; Luciński et al., 2024). It was also proven that Egy2 protease participates in the regulation of chloroplast gene expression, probably via releasing from thylakoid membrane pTAC10 and pTAC16 proteins, which interact with the plastid-encoded RNA polymerase complex (Adamiec et al., 2018; Luciński et al., 2024).

There is quite a lot of literature reports showing that intramembrane chloroplast S2P proteases, namely, EGY1 and EGY2, play an important role in shaping and maintaining the correct stoichiometric relations between the polypeptides building the main photosynthetic complexes of the *A. thaliana* thylakoid membranes, in particular, photosystem II (PS II) and its main light-harvesting antenna—LHC II (Chen et al., 2005, 2016, Yu et al., 2016; Adamiec et al., 2018; Qi et al., 2020; Adamiec et al., 2021a; Luciński et al., 2024). The lack of these proteases is also associated with increased sensitivity of plants to photoinhibition (Adamiec et al., 2018, 2021a, Luciński et al., 2024). The latter effect is also observed in the absence of the EGY3 pseudoprotease (Adamiec et al., 2020), the absence of which is also associated with increased production of reactive oxygen species by chloroplasts of plants exposed to the stress of high temperature and high light intensity (Adamiec et al., 2022).

S2P2 remains the least known chloroplast S2P protease. The protein was considered to be involved in cold stress response (Wang et al., 2016); however, during the *A. thaliana* development, the gene encoding this protein displayed high expression levels in cotyledons and rosette leaves of *A. thaliana* (Winter et al., 2007), which indicates on its possible role in maintaining proper chloroplast function. There is however no data concerning the chloroplast status in plants deprived of the S2P2 protease. Therefore, we decided to analyze in detail the possible role that S2P2 plays in chloroplasts. We experimentally investigated the intra-chloroplast localization of this protease, analyzed how the lack of S2P2 in *A. thaliana* affects the chloroplast proteome, and considered the potential role of S2P2 protease in the correct functioning of photosystem II under both non-stressful light intensity and under photoinhibitory conditions.

## Materials and methods

### Plant material and growth conditions

Wild-type (WT) and *A. thaliana* (L.) Heynh (ecotype Columbia) mutant plants were grown on sphagnum peat moss and wood pulp in 42-mm Jiffy peat pellets (AgroWit, Przylep, Poland) under long-day conditions (16 h of light and 8 h of darkness) at an irradiance of 110 µmol m^−2^ s^−1^, a constant temperature of 22°C, and a relative humidity of 70%.

*A. thaliana* seeds with a T-DNA insertion in the *S2P2* gene (*At1g05140*), described as SALK_046599C (*s2p2-1*) and SALK_071288 (*s2p2-2*), were obtained from NASC (Nottingham Arabidopsis Stock Centre, Nottingham, UK). Homozygosity of the T-DNA insertion within the analyzed gene was confirmed by PCR. The following primers were used for *s2p2-1* and *s2p2-2* lines analysis:

forward: 5′-TTTTCTTTTCTCCGCATTTTG-3′
reverse: 5′-TAAAAAGTGACCGGTCTCGTG-3′
T-DNA insertion (LB): 5′-GCGTGGACCGCTTGCTGCAACT-3′

### Isolation and fractioning of chloroplasts

All stages of the procedure were carried out in a cold room under a green light according to Chen et al. (2016); Moyet et al. (2017), and our previous work (Adamiec et al., 2020) and were preceded by an 8-h darkening of *A. thaliana* plants. The collected leaves were immediately homogenized in an ice-cold homogenization buffer (50 mM HEPES–KOH, pH 7.8, 330 mM sorbitol, 10 mM EDTA, 5 mM NaCl, 5 mM MgCl_2_, 5 mM sodium ascorbate, and 0.2% (w/v) BSA) in the 1:10 proportion (w/v; mass of leaves: volume of buffer). The homogenate was filtered through a 100 - μm mesh filter and centrifuged at 1,000*g* for 5 min at 4°C. The obtained green pellet was resuspended again in the homogenized buffer and centrifuged as before. Finally, the pellet containing the crude intact chloroplast fraction was resuspended in 40-mL homogenization buffer and loaded on the top of the previously formed Percoll gradient (50% Percoll in the homogenization buffer centrifuged at 38,700*g* for 55 min at 4°C) and centrifuged at 13,300*g* for 10 min at 4°C. The intact chloroplasts were collected from the dark-green band in the lower part of the gradient, resuspended in 200 mL of homogenization buffer, and centrifuged at 2,000*g* for 2 min at 4°C. The purified intact chloroplasts were lysed in lysis buffer (MOPS–NaOH pH 7.8, 4mM MgCl_2_, 1 mM PMSF, 1 mM benzamidine, and 0.5 mM ε-amino caproic acid), loaded on the top of the sucrose gradient (10 mM MOPS–NaOH pH 7.8, 4 mM MgCl_2_, and 0.6 and 0.93 M sucrose), and centrifuged at 70,000*g* for 1 h at 4°C. The envelope membranes were present as a yellow band at the 0.93/0.6-M sucrose interface. The thylakoid membranes were visible as the dark-green band at the bottom of the tube. The stromal proteins were on top of the gradient. To purify and concentrate the envelope membranes, the yellow band containing the envelope was diluted in fourfold lysis buffer and centrifuged at 110,000*g* for 1 h at 4°C. The pellet containing the envelope membranes was resuspended in minimum-volume lysis buffer and stored in liquid nitrogen. Similarly, the thylakoid membranes were also resuspended in the lysis buffer and stored in liquid nitrogen.

### Protein isolation and determination of protein concentration

Total protein was isolated from 100 mg of *A. thaliana* leaf tissue using protein extraction buffer (PEB, Vannas, Agrisera), according to the manufacturers’ instructions. The concentration of the extracted protein was determined using the modified Lowry (Lowry et al., 1951) method with a Lowry DC kit (Bio-Rad, Hercules, CA, USA).

### SDS-PAGE and immunoblotting

SDS-PAGE was performed according to Laemmli (1970) in 12% polyacrylamide gels with the addition of 6 M urea, followed by the transfer of proteins from the gel to a PVDF membrane with a pore size 0.2 µm (Bio-Rad, USA). The standard Western blot procedure was then performed, according to our previous work (Adamiec et al., 2018, 2021a). The PVDF membranes were blocked with 4% BSA (BioShop, Burlington, Canada) and then incubated with specific primary antibodies for 90 min. After incubation with secondary antibodies (Agrisera, Vannas, Sweden), the relevant bands were visualized using the ChemiDoc™ MP Imaging System (Bio-Rad, USA) following 5-min incubation of the PVDF membrane with Clarity Western ECL Substrate (Bio-Rad, Hercules, CA, USA). According to our previous work (Luciński et al., 2024), only blots with a linear relationship between the strength of the signal and the amount of protein used were considered.

### Antibodies

The specific polyclonal anti-S2P2 antibodies were custom produced in rabbits by Davids Biotechnologie GmbH (Germany), using the highly specific synthetically obtained antigen (AA sequence: DNDPDSDIPVDDRNLLKNR). Other primary antibodies (anti-H3, anti-ACT, anti-COXII, anti-TOC75, anti-PsbA, anti-PsbC, anti-PsbD, and anti-Lhcbs) as well as secondary antibodies were purchased from Agrisera AB (Vannas, Sweden).

### Blue native gel electrophoresis

Blue native gel electrophoresis was performed according to Wittig et al. (2006); Chen et al. (2016); Liu & Last (2017), and our previous work (Adamiec et al., 2018, 2021a). The thylakoid membranes corresponding to 15 µg of chlorophyll were centrifuged and resuspended in ice-cold resuspension buffer (25 mM Bis–Tris–HCl pH 7.0, 20% (v/v) glycerol) to a final concentration of chlorophyll equal to 1.0 mg mL^−1^. The solubilization of the thylakoid membranes was performed in darkness for 10 min at 4°C with gentle mixing using 2% (w/v) *n*-dodecyl-β-maltoside in the resuspension buffer. Traces of insoluble materials were separated by centrifugation at 18,000*g* for 20 min at 4°C. The supernatant was mixed with 0.1 volumes of sample buffer (100 mM Bis–Tris–HCl pH 7.0, 0.5 M amino-*n*-caproic acid, 30% (w/v) sucrose, 50 mg mL^−1^ Serva Blue G) and loaded onto native precasted, gradient 4%–14% acrylamide gels (Bio-Rad, USA). Electrophoresis was performed with the use of the cathode buffer (50 mM tricine, 7.5 mM imidazole, 0.02% (w/v) Coomassie blue G 250, pH ~7.0) and the anode buffer (7.5 mM imidazole, pH ~7.0) for approximately 6 h at 4°C with the voltage increasing from 80 V to 200 V. To facilitate detection of the less abundant proteins, the cathode buffer was replaced with a 10-times diluted cathode buffer after the front reached approximately three-quarters of the total distance. Finally, to reduce the blue background, the gel was rinsed in water overnight at room temperature.

### Determination of chlorophyll and carotenoid concentrations

The chlorophyll and carotenoid concentrations were measured using a DMSO assay (Hiscox and Israelstam, 1979 *). The following* equations were used to determine the concentrations (µg/ml) of chlorophyll *a* (Chl *a*), chlorophyll *b* (Chl *b*), and total carotenoids (C *x+c*), defined as the sum of xanthophylls (x) and carotenes (c) (Nayek et al., 2014).


$$\text{Chl }a=12.47{\text{ A}}_{665}–3.62{\text{ A}}_{649}$$

$$\text{Chl }b=25.06{\text{ A}}_{649}–6.5{\text{ A}}_{665}$$

$$\text{C }x+c=\left(1000{\text{ A}}_{470}–1.29\text{ Chl }a–53.78\text{ Chl }b\right)/220$$

### Chlorophyll *a* fluorescence measurement

Chlorophyll *a* fluorescence measurements were conducted using FMS1 (Photon System Instruments, Brno, Czech Republic) run by Modfluor software, according to the Genty et al. (1989) protocol. The leaves were dark-adapted for 30 min before each measurement. The minimum fluorescence yield (F_0_) was established at the beginning of measurement. The maximum quantum yield of PSII (F_v_/F_m_) and quantum efficiency of open centers in the light (F_v_′/F_m_′) were calculated according to Genty et al. (1989). The applied actinic light intensity was equal to the irradiance prior to dark adaptation: 110 µmol m^−2^ s^−1^. The photochemical quenching (qP), photochemical yield of photosystem II (ΦPSII), and non-photochemical quenching parameter (NPQ) were calculated according to Maxwell and Johnson (2000). There were 30 plants from each variant (WT, *s2p2-1*, and *s2p2-2*) measured in each replicate.

### Time-course photoinhibition and the recovery assays

A time-course analysis was performed according to Liu and Last (2017) and our previous work (Adamiec et al., 2018, 2020). The detached *A. thaliana* leaves of the WT and both *s2p2* mutant lines were infiltrated with 1 mM lincomycin (Sigma-Aldrich, USA) solution or water and illuminated with 1,000 μmol·m^−2^ s^−1^ for 4 h. The F_v_/F_m_ parameter was measured every hour after 30 min of dark adaptation of the leaves. For the time-course recovery, the detached leaves, which reached approximately 70%–75% of the initial F_v_/F_m_ value during exposure to the photoinhibitory irradiance, were shifted to normal growth light (110 μmol·m^−2^ s^−1^). During the recovery, the F_v_/F_m_ was measured every hour until no further changes in the F_v_/F_m_ were observed.

### Statistical analysis

Differences in the measured parameters were analyzed for statistical significance using one-way ANOVA. Means were regarded as significantly different at P< 0.05.

## Results

### *S2p2* T-DNA insertion mutants

**Figure 1:**
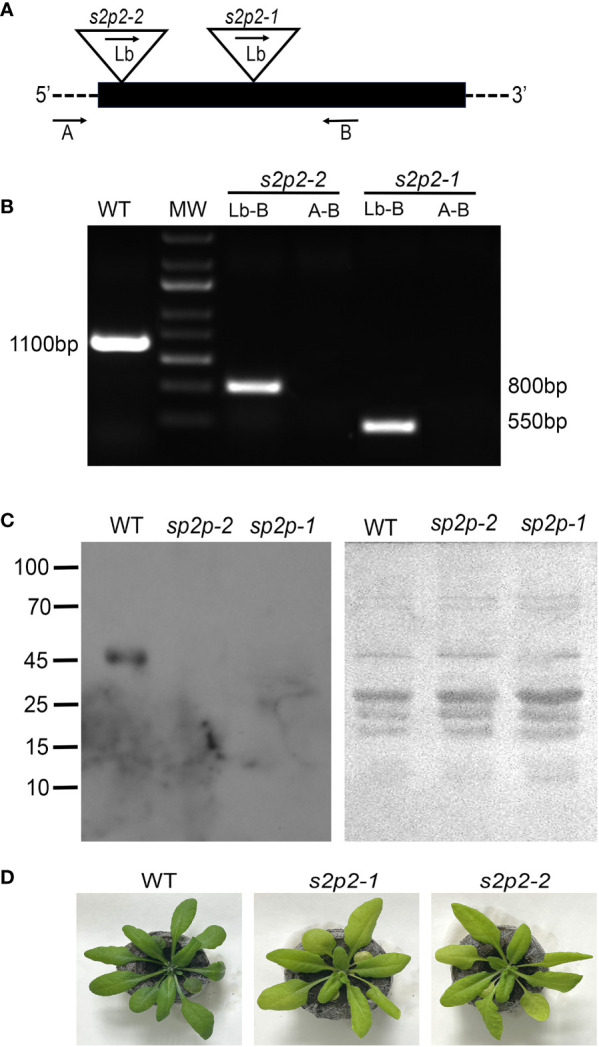
Identification of *s2p2* mutants. **(A)** Schematic diagram of the *A. thaliana* S2P2 gene. The black box represents exons. The triangles show the locations of T-DNA insertions. The arrows mark the annealing sites of the primers used for PCR analysis. **(B)** Confirmation of the homozygosity of the *s2p2-1* and *s2p2-2* mutant lines. Amplification was performed using the A, B, and Lb primers as indicated in A. **(C)** Immunoblot analysis of the S2P2 protease presence in the wild-type (WT) and *s2p2-1* and *s2p2-2* mutants. Samples of total protein (5 μg) were resolved by SDS-PAGE, electrotransferred to PVDF membranes and immunostained with the anti-S2P2 antibody. Before hybridization, the PVDF membranes were stained with Ponceau S to control loading. **(D)** Pictures of the 4-week-old WT, *s2p2-1*, and *s2p2-2* mutant line plants.

The physiological role of the S2P2 protease was studied using two commercially available lines with T-DNA insertion in the At1g05140 gene, encoding the S2P2 protein: SALK_046599 described as *s2p2-1* and SALK_071288 described as *s2p2-2.* In both lines, T-DNA insertion is located within the coding sequence (**Figure 1A**). The homozygosity of both mutant lines was confirmed by the PCR method using specific primers (**Figure 1B**). The absence of the S2P2 protease in both the *s2p2-1* and *s2p2-2* lines was confirmed by immunoblot analysis with the use of specific anti-S2P2 antibodies (**Figure 1C**). Visible phenotypic changes in both mutant lines resulted in a slightly less intense green color of the leaves (**Figure 1D**). This effect is most likely related to the reduced content of chlorophylls and in *s2p2* mutants (**Table 1**). Otherwise, no visible deformations were observed.

**Table 1:** Comparison of chlorophyll and carotenoid content in leaves [μg g^−1^ fresh weight] and chlorophyll *a* fluorescence parameter in the wild-type (WT) plants and *s2p2* mutant lines (*s2p2-1* and *s2p2-2*) in the non-stressed light conditions.

	WT	*s2p2-1*	*s2p2-2*
Total chlorophyll	860 ± 89	511^*^ ± 47	414^*^ ± 39
Chlorophyll *a*	556 ± 41	293^*^ ± 20	302^*^ ± 19
Chlorophyll *b*	304 ± 17	218^*^ ± 16	218^*^ ± 18
Chlorophyll a/b	1.83 ± 0.15	1.34^*^ ± 0.11	1.38^*^ ± 0.12
Carotenoids	123 ± 13	347^*^ ± 28	385^*^ ± 34
F_0_	106 ± 11	168^*^ ± 21	159^*^ ± 17
F_v_/F_m_	0.831 ± 0.02	0.812 ± 0.01	0.826 ± 0.01
qP	0.901 ± 0.03	0.898 ± 0.04	0.900 ± 0.03
NPQ	0.116 ± 0.03	0.276^*^ ± 0.02	0.289^*^ ± 0.04
ΦPSII	0.684 ± 0.02	0.678 ± 0.02	0.681 ± 0.03

“±” indicates the SD calculated from analysis of three biological replicates (30 plants each); “*” indicates statistically significant differences between WT and individual mutants.

### Subcellular localization of S2P2 protein

**Figure 2:**
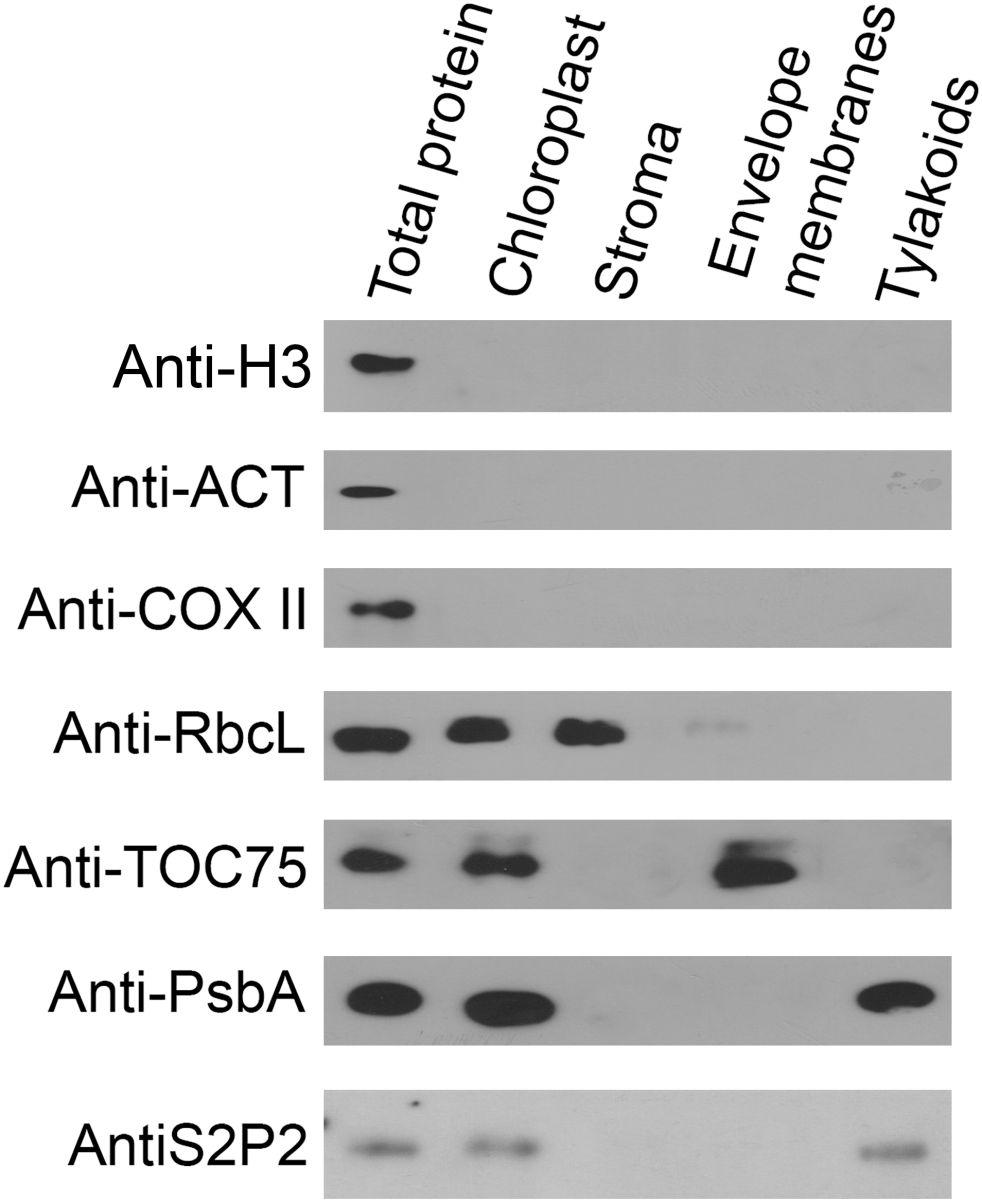
Immunolocalization of S2P2. The total protein from leaves and the protein extract from intact chloroplasts, stroma, chloroplasts’ envelope, and thylakoid membranes were separated by SDS-PAGE, transferred onto the PVDF membrane, and immunologically analyzed with specific primary antibodies against histone H3 (anti-H3), actin (anti-ACT), cytochrome c oxidase (anti-COX II), Rubisco large subunit (anti-RbcL), PsbA (anti-PsbA), TOC75 (anti-TOC75), and S2P2 (anti-S2P2).

The chloroplast localization of S2P2 was postulated based on amino acid sequence analysis and large-scale analysis of the chloroplast proteome (Zybailov et al., 2008; Adamiec et al., 2017). However, the exact location of this protein within chloroplasts was still unknown. Therefore, the intra-chloroplast localization of S2P2 was investigated by immunoblot experiments (**Figure 2**). The total leaf protein extract as well as highly purified chloroplasts and fractions of stroma, envelope, and thylakoid membrane proteins were tested for the presence of S2P2 protein. To verify the purity of the chloroplast fraction, immunoblot tests were performed using specific antibodies against protein markers of the nucleus, cytoplasm, and mitochondria: cytochrome c oxidase (anti-COXII), which is an element of the respiratory chain in the inner mitochondrial membrane, was used as a mitochondrial marker. The possible contamination of chloroplasts with the cytosol fraction was examined with the antibody against actin (anti-ACT), and the presence of nuclear proteins was verified with the antibody against H3 histone protein (anti-H3). The pure chloroplast fraction was further fractionated into stroma, envelope, and thylakoid membranes. The anti-RbcL, anti-TOC75, and anti-PsbA antibodies were used as stroma, envelope, and thylakoid membrane markers, respectively. The immunoblot analysis revealed that the chloroplast fraction and each chloroplast subfraction, i.e., stroma, envelope, and thylakoid membranes, were free from contamination. The results obtained also confirm the chloroplast localization of S2P2 and indicate the intra-chloroplast localization of this protease protein in the thylakoid membrane (**Figure 2**).

**Figure 3:**
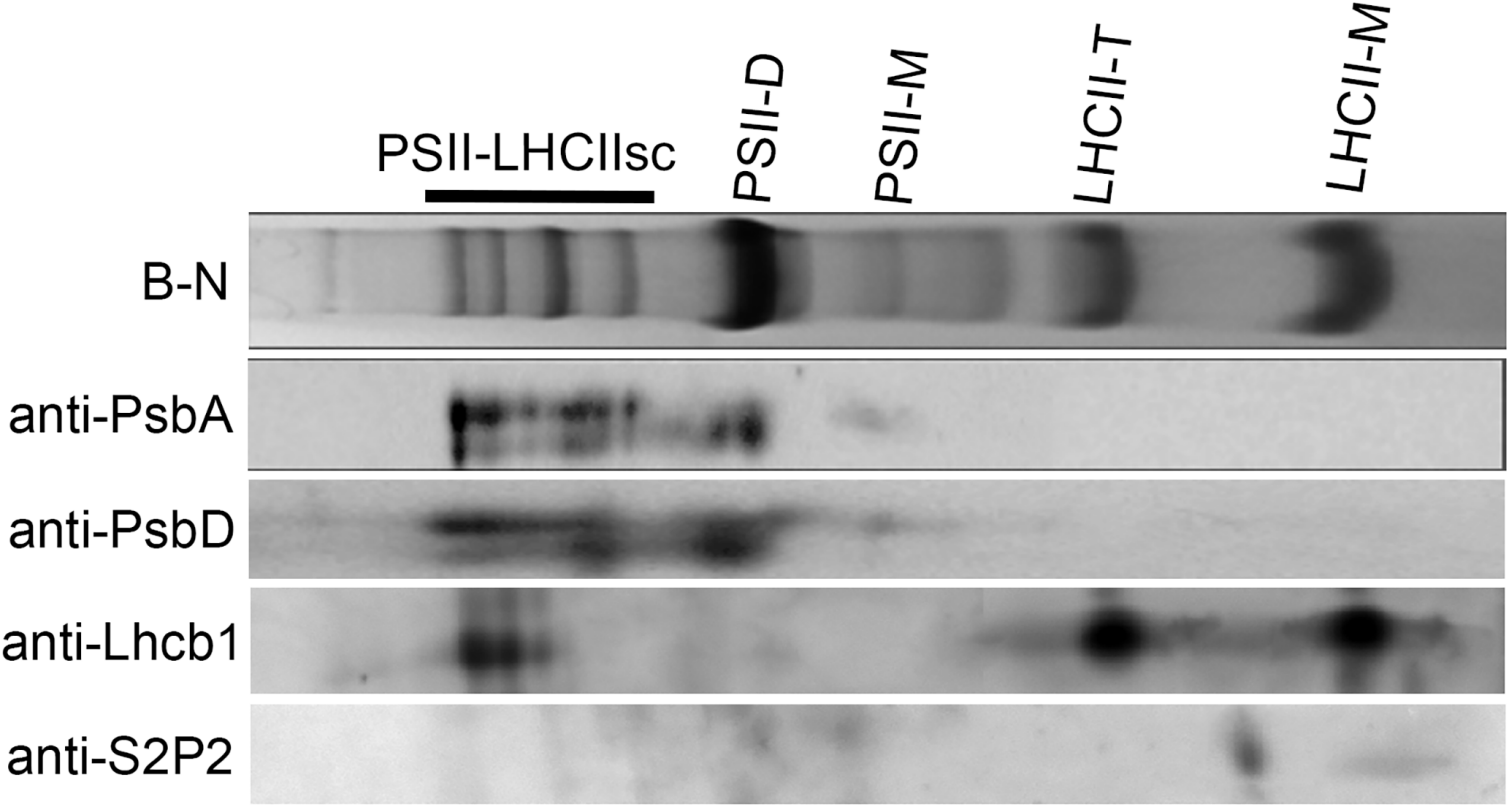
Analysis of S2P2 co-migration with thylakoid membrane complexes. The chlorophyll–protein complexes were prepared from thylakoid membranes of WT *A. thaliana* leaves and fractioned by two-dimensional blue native/SDS-PAGE and detected by specific antibodies as in the figure. PSII-LHCIIsc–PSII-LHCII supercomplexes, PSII-D–PSII dimer, PSII-M–PSII monomer, LHCII-T–LHCII trimer, and LHCII-M–LHCII monomer.

Due to the localization of S2P2 within the thylakoid membranes, an attempt was also made to determine the possible co-migration of S2P2 with one of the thylakoid photosynthetic complexes. For this purpose, the blue native electrophoresis was applied to the thylakoid pigment–protein complexes separation and then the SDS-PAGE was used for the second-dimension separation. Finally, the proteins were transferred to PVDF membranes and the PsbA, PsbD, Lhcb1, and S2P2 polypeptides were visualized using the western blot procedure. As expected, PsbA and PsbD polypeptides were located in the area corresponding to the location of the PSII–LHCII supercomplexes as well as the PS II dimer and PS II monomer. In turn, the Lhcb1 polypeptide was identified in the area corresponding to the location of the trimeric and monomeric forms of the LHC II complex. A protein spot corresponding to S2P2 was visible between the trimeric and monomeric forms of LHC II, which suggests the lack of co-migration of S2P2 with any of the thylakoid photosynthetic complexes (**Figure 3**).

### The quantitative comparison of thylakoid membrane complexes

**Figure 4:**
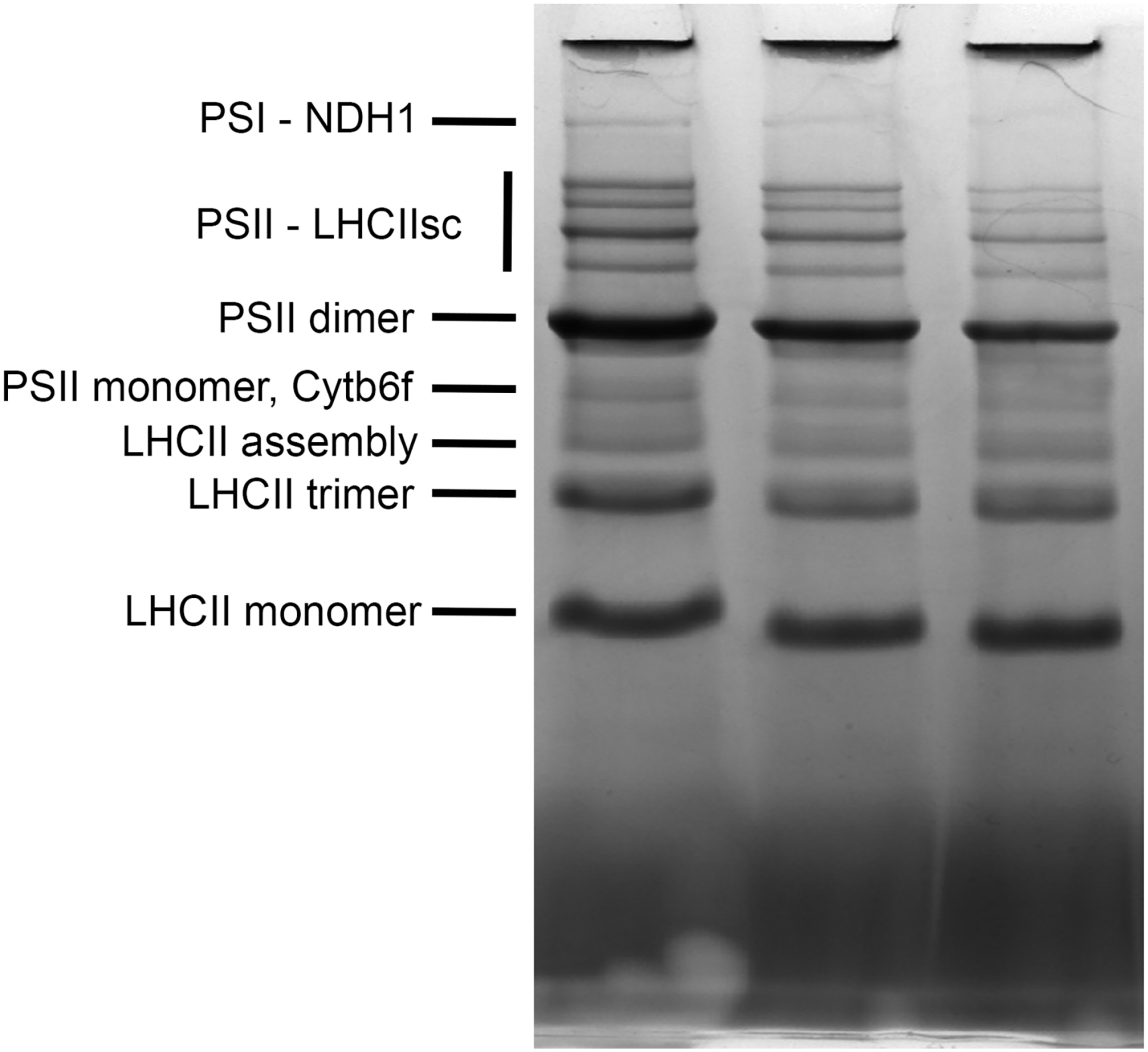
Blue native PAGE of thylakoid membrane complexes. Thylakoid membranes were isolated from wild-type (WT) plants and *s2p2-1* and *s2p2-2* mutant lines of *A. thaliana* and solubilized with 1% (w/v) n-dodecyl-β-maltoside. Thylakoid membrane complexes (15 μg) were loaded onto 4%–14% gradient, native, precasted gel, and separated by blue native PAGE.

The blue native electrophoresis technique was applied to the quantitative comparison of thylakoid membrane complexes’ abundance in *s2p2* mutant lines and WT plants, growing under non-stressful light conditions (**Figure 4**). The comparative densitometric analysis (mutant lines vs. WT plants) revealed significant differences in the accumulation of individual thylakoid complexes (**Table 2**). In both *s2p2* mutant lines, the intensity of the band corresponding to the PSI–NDH1 complex was reduced by approximately 46% compared with the WT plants in the *s2p2-1* and as much as 73% in the *s2p2-2* line. In both mutant lines the intensities of the bands representing PS II supercomplexes were reduced by approximately 30% compared with the WT plants. This was accompanied by a significant decrease in the content of the PS II dimer and monomer by approximately 30% and 20%, respectively. The intensity of the bands corresponding to the LHC II assembly complexes and LHC II in trimeric form were also significantly reduced in *s2p2* mutant lines; however, the LHC II monomer did not show statistically significant changes in amounts (**Table 2**).

**Table 2:** The results of densitometry of thylakoid membrane complexes isolated from WT plants and *s2p2* lines.

Band name		WT (%)	*s2p2-1* (%WT ± SD)	*s2p2-2* (%WT ± SD)
PSI-NDH1		100	54* ± 12	27* ± 18
PSII-LHCIIsc	C_2_S_2_M_2_	100	68* ± 7	55* ± 10
	C_2_S_2_M	100	72* ± 8	57* ± 11
	C_2_S_2_	100	63 *± 6	46* ± 11
	C_2_S	100	76* ± 8	54* ± 9
PSII dimer		100	72* ± 8	65* ± 8
PSII monomer, Cytb6f		100	80 ± 7	81* ± 8
LHCII assembly		100	76* ± 7	82 ± 9
LHCII trimer		100	68* ± 8	73* ± 7
LHCII monomer		100	79 ± 9	88 ± 10

“±” indicates SD, and “*” indicates statistically significant differences between WT and individual *s2p2* mutant lines. Samples from three biological replicates were analyzed.

### The abundance of PSII apoproteins in *s2p2* mutant lines

**Figure 5:**
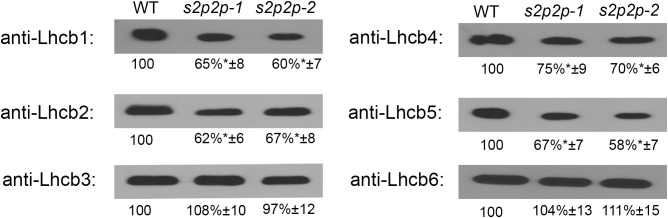
Immunoblot analysis of the levels of Lhcb1-6 polypeptides in wild-type (WT) plants and *s2p2-1* and *s2p2-2* mutant lines. Total proteins (2 μg) from each sample were subjected to immunoblotting analysis with specific primary antibodies. GelixOne software was used for blot quantification. The individual polypeptide content of the mutant lines was quantified as a percentage of the antibody signal strength in the WT (100%). “±” indicates the SD calculated from the analysis of samples from the four biological replicates. The asterisks indicate statistically significant differences between the WT and individual mutant line.

**Figure 6:**
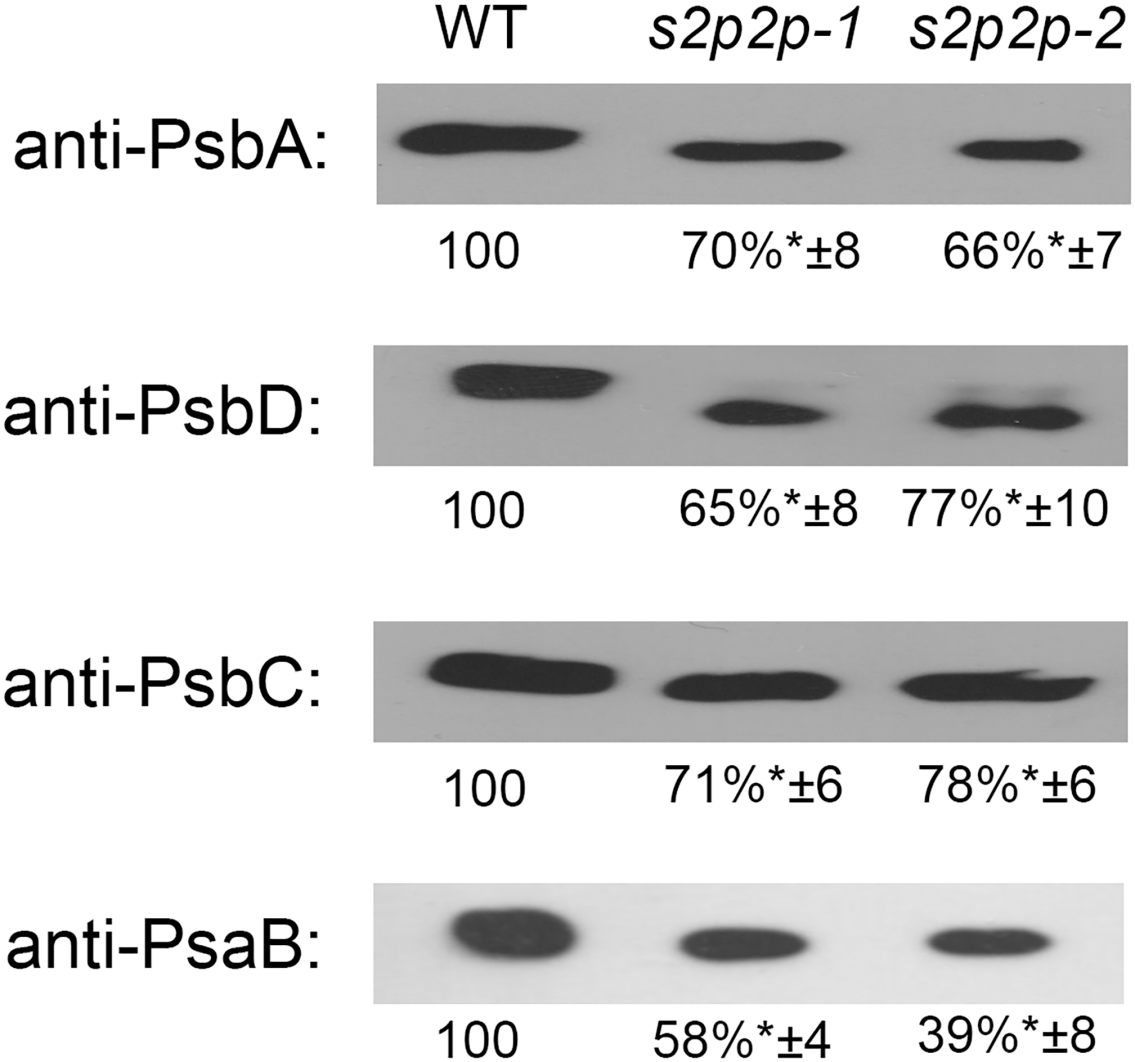
Quantification of PsbA, PsbD, PsbC, and PsaB apoproteins in the wild-type (WT) plants and *s2p2-1* and *s2p2-2* mutant lines. Samples containing 2 μg of total proteins were separated by SDS-PAGE, transferred to PVDF membranes, and subjected to immunoblot analysis with specific primary antibodies. Quantifications of the blots was performed with the use of GelixOne software. Immunoblot signal strength in mutant lines was normalized against signal strength in the WT (100%). The experiment was performed in four biological replicates. “±” indicates the SD, and the asterisks indicate statistically significant differences between the WT and individual mutant line.

Immunoblot analysis of the selected PSII–LHCII apoproteins revealed some important changes in the accumulation levels of the nuclear-encoded apoproteins Lhcb1–Lhcb6 (**Figure 5**) as well as chloroplasts’ encoded PS II and PS I core proteins, namely, PsbA, PsbC, PsbD, and PsaB (**Figure 6**).

The level of two LHCII polypeptides, namely, Lhcb1 and Lhcb2, in the *s2p2-1* and *s2p2-2* mutants, measured in the leaves of 4-week-old plants, was significantly lower than in the WT plants (**Figure 5**). The content of Lhcb1 decreased to 65% and 60%, whereas that of Lhcb2 reached 62% and 67% of the WT level for *s2p2-1* and *s2p2-2*, respectively. The level of Lhcb3 in the mutants was similar to that in WT.

Among the polypeptides that build the so-called minor peripheral light-harvesting complexes, the Lhcb6 polypeptide remained in the mutant at a level identical to that in WT. However, the Lhcb4 and Lhcb5 polypeptides, which constitute the apoprotein moiety of CP29 and CP26, respectively, showed significantly lower levels (**Figure 5**). The Lhcb4 level decreased to 75% and 70% in *s2p2-1* and *s2p2-2*, respectively, whereas Lhcb5 showed a slightly deeper decline to levels of 67% and 58% of WT values (*s2p2-1* and *s2p2-2*, respectively).

PS II core proteins—PsbA, PsbD, and PsbC—also showed significantly lower levels when comparing *s2p2* mutants with WT plants (**Figure 6**). Namely, the levels of all these proteins were reduced, in both mutant lines, by approximately 30% compared with WT plants. This result is consistent with the measured values of chlorophyll *a* fluorescence and the increased sensitivity of *s2p2* mutants to photoinhibition.

A stronger decrease was observed in the case of the PsaB polypeptide, which is the PS I core protein. The level of PsaB in the *s2p2-1* mutant line was approximately 58% compared with the level of this polypeptide in WT plants, whereas in the *s2p2-2* line, the PsaB decrease was even more pronounced, to 38% of the WT value (**Figure 6**).

### The pigment content and chlorophyll *a* fluorescence

The pigment content (chlorophylls and carotenoids) in leaves of *s2p2-1* and *s2p2-2* mutant lines was investigated, and significant differences were revealed between both mutant lines and WT plants (**Table 1**). A statistically significant decrease in chlorophyll *a* and chlorophyll *b* content was observed, and this reduction was accompanied by a reduction in the Chl *a*/*b* ratio. The reduced level of chlorophylls resulted in a less intense green color of the leaves of both mutant lines. On the contrary, the carotenoid content in both *s2p2* mutant lines was significantly higher than in the leaves of WT plants (**Table 1**).

Chlorophyll *a* fluorescence was also investigated. The PAM fluorescence technique we used included measurements of minimum fluorescence yield (F_0_), the maximum quantum yield of PSII (Fv/Fm), and quantum efficiency of PS II electron transport in the light (ΦPS II), photochemical quenching (qP), and non-photochemical quenching (NPQ). The *s2p2* mutant plants grown under non-stressful light conditions (110 μmol m^−2^ s^−1^) revealed a higher value of the F_0_ parameter, which reflects the rate constant of energy trapping by PS II centers. The F_v_/F_m_ and qP parameters as well as ΦPS II in both mutant lines reached values similar to WT plants. However, in *s2p2* mutants, we observed an increased value of the non-photochemical quenching parameter (NPQ), which reflects changes in the efficiency of heat dissipation (**Table 1**).

**Figure 7:**
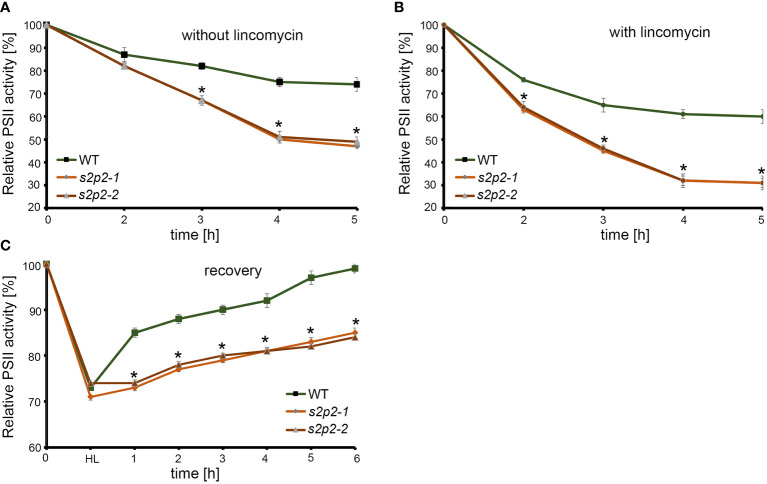
The time-course changes of maximum PS II efficiency (F_v_/F_m_). Leaves were detached from 4-week-old plants grown under 110 μmol photons m^−2^ s^−1^ and treated with water **(A)** or 1 mM lincomycin **(B)** and placed under 1,000 μmol photons m^−2^ s^−1^ for 5 h. F_v_/F_m_ was measured after 2 h, 3 h, 4 h, and 5 h of exposure to high light. **(C)** To measure the changes of F_v_/F_m_ in the recovery phase, leaves were exposed to 1,000 μmol photons m^−2^ s^−1^ until the F_v_/F_m_ parameter reached approximately 70%–75% of the maximum initial value (WT, *s2p2-1*, and *s2p2-2* leaves) and transferred again to 110 μmol photons m^−2^ s^−1^. The F_v_/F_m_ measurements were taken every hour until full recovery of PS II in WT plants was observed. The asterisks indicate statistically significant differences between the WT and individual mutant line.

Apart from analyzing the functional parameters of PS II under non-stressful light conditions, we also analyzed the sensitivity of *s2p2* mutant lines to photoinhibition (**Figure 7**). The photoinhibition means the light-dependent irreversible inactivation of PS II, which can be restored via the degradation and *de novo* synthesis of the PsbA protein (Tyystjärvi and Aro, 1996). The rate of decrease of the maximum quantum yield of PS II (F_v_/F_m_) under photoinhibitory irradiance (1,000 μmol m^−2^ s^−1^) in both *s2p2* mutant lines was significantly deeper and faster than in WT plants, both in the presence and in the absence of lincomycin (**Figures 7A, B**). Lincomycin is an inhibitor of chloroplast protein synthesis, which arrests translation of the *PSBA* gene product. Furthermore, the time-course experiment showed that after the shift to normal growth irradiance (110 μmol m^−2^ s^−1^), during the recovery phase, the F_v_/F_m_ parameter increases significantly slower in *s2p2* mutant lines than in WT plants (**Figure 7C**).

## Discussion

S2P2 is a nuclear-encoded protein that belongs to the zinc-containing intramembrane protease family, specifically the S2P family (Adamiec et al., 2017). So far, six genes encoding proteins belonging to this family have been identified in the *A. thaliana* genome: EGY1, EGY2, EGY3, S2P2, ARASP, and the product of the *At4g20310* gene (Chen et al., 2005; Bölter et al., 2006; Zybailov et al., 2008; Che et al., 2010; Chen et al., 2012). Except for the product of the *At4g20310* gene, the remaining proteins are located within chloroplasts. Much is already known about the significance and possible functions of EGY1 (e.g., Chen et al., 2005, 2016; Yu et al., 2016; Qi et al., 2020; Adamiec et al., 2021a; Adamiec et al., 2021b) and EGY2 proteases (Chen et al., 2012; Adamiec et al., 2018; Luciński et al., 2024), as well as EGY3, which, due to the lack of the characteristic zinc-binding motif (HExxH), lacks proteolytic activity and is classified as a so-called pseudoprotease (Adamiec et al., 2020, 2022). However, knowledge about the physiological functions of S2P2 is extremely limited. The chloroplast localization of this protein was postulated based on the analysis of large-scale chloroplast proteome studies (Zybailov et al., 2008). Nevertheless, no studies are focusing on the intra-chloroplast localization of S2P2. Here, we conducted a detailed western blot analysis, using specific anti-S2P2 antibodies. A differential centrifugation technique using the Percoll and sucrose gradients allowed the obtaining of pure chloroplasts’ fractions of stroma, envelope, and thylakoid membrane. Specific antibodies against marker proteins for each fraction were used to identify and confirm the purity of individual fractions. We previously successfully employed this technique to study the localization of the EGY3 protein (Adamiec et al., 2020). The results not only confirmed the chloroplast localization of S2P2 but also demonstrated the precise localization of this protease within the thylakoid membrane. However, S2P2 protein has not been shown to co-migrate with any of the large photosynthetic complexes present in thylakoid membranes, i.e., PS II, PS I, cytb6/f, or LHC II, during blue native electrophoresis. Therefore, it seems rather unlikely that S2P2 will form complexes with one of these particles.

The physiological role of S2P2 protease was investigated using two homozygous mutant lines, referred to as *s2p2-1* (SALK_046599) and *s2p2-2* (SALK_071288). The homozygosity of both lines was confirmed using PCR, and the absence of the S2P2 protein was verified using western blot analysis with specific anti-S2P2 antibodies.

Using the blue native gel electrophoresis technique, the impact of the lack of S2P2 on the composition of the main photosynthetic complexes in the thylakoid membranes was examined. Both *s2p2* mutant lines exhibited a lower abundance of PS II dimers and LHC II trimers. Moreover, a decrease was observed in more associated forms of these complexes. The PS II–LHC II supercomplexes consist of PS II core complex homodimers (C_2_) linked with Lhcb4, Lhcb5, and Lhcb6 proteins, which form a minor peripheral antenna system and play a crucial role in binding the major light-harvesting complexes (LHC II). The LHC II complexes, in turn, are homo- or heterotrimers composed of Lhcb1, Lhcb2, and Lhcb3 proteins, which may be associated with the PS II core complex with different affinities (Cao et al., 2018). Two of the LHC II trimers strongly associate with homodimers and are described as C_2_S_2_. Additionally, two more LHC II complexes can bind to the PS II core with moderate affinity. The complexes containing all four LHC II trimers are named C_2_S_2_M_2_ (Horton et al., 2008; Nosek et al., 2017). In the thylakoid membranes isolated from *s2p2* mutants, the abundance of all forms of PS II–LHC II supercomplexes was lower than in WT (wild-type) plants.

Moreover, the analyses of blue native gels revealed that the content of the PS I–NDH1 complex was also significantly reduced in *s2p2* mutants. The decrease in the content of PS I–NDH1 was even clearly greater than in the case of PS II and PS II–LHC II supercomplexes, which was particularly visible in the *s2p2-2* line. A significant decrease of the PS I particles in *s2p2* mutants may be important for the functioning of the linear and cyclic electron transport chain, and thus the ability of the mutants to produce NADPH and ATP. PS I is a key site determining the balance of ATP/NADPH production (Yamori and Shikanai, 2016) and plays an important role in the regulation of photosynthetic electron transport, which is necessary for the effective response of chloroplasts to fluctuations in light intensity (Ji et al., 2022).

The above observations taken together suggest that the absence of S2P2 has an impact on the organization and abundance of the photosynthetic complexes in the thylakoid membranes, potentially affecting the efficiency of the photosynthetic process.

Quantitative changes in the content of individual thylakoid complexes observed in blue native gels were confirmed in analyses of chlorophyll content and the amounts of specific proteins building up PS I, PS II, and LHC II. In both *s2p2* mutant lines, a distinctly lower content of chlorophyll *a* and chlorophyll *b* was noticed compared with WT plants, as well as a significantly lower amount of the PsbA, PsbD, and PsbC as well as PsaB apoproteins. In accordance with the widely accepted model PsaB forms, together with PsaA, the PS I reaction center heterodimer (Busch and Hippler, 2011) and PsbA, PsbD, and PsbC are the crucial parts of the PS II core structure. According to the atomic resolution structural data, these three polypeptides occur in PS II in a stoichiometric ratio of 1:1:1. PsbA and PsbD form the reaction center heterodimer, closely associated with PsbC, which constitutes an inner light-harvesting antenna of PS II (Luciński and Jackowski, 2006). The lower abundance of these polypeptides indicates the inability to form a sufficient number of functional PS II particles, a fact further confirmed by images from blue native gel electrophoresis. The decrease was also observed in the content of Lhcb1 and Lhcb2 proteins, whereas the abundance of the third component building LHC II–Lhcb3 complexes remained unchanged. The Lhcb1 and Lhcb2 proteins are major proteins forming LHC II complexes and constitute approximately 89% of LHC II protein content (Jackowski et al., 2001; Luciński and Jackowski, 2006; Luciński and Jackowski, 2013); thus, these results correlate well with a decrease in free LHC II trimers and PS II–LHC II supercomplexes in analyzed mutant lines. Moreover, the decreased abundance of Lhcb4 and Lhcb5 forming minor peripheral antenna was also observed. Simultaneously, *s2p2* mutants were characterized with a decreased chlorophyll *a/b* ratio. This parameter is a good indicator of antenna size since Chl *a* is present in both the PSII core and LHC II antenna whereas Chl *b* occurs mainly in the peripheral antenna (Tanaka et al., 2001; Hoober et al., 2007). The obtained results indicate that the absence of S2P2 leads to a decrease in the overall pool of both PS II core and LHC II complexes. Simultaneously, there is an increase in the number of antennas per reaction center of PS II.

Changes in the stoichiometry of PS II core and LHC II proteins, along with the decrease in chlorophyll content, are factors that can negatively impact the functioning of PS II. The chlorophyll *a* fluorescence measurement was used to determine the fundamental parameters describing the functional state of PS II. In non-stressful light conditions, the observed stoichiometric changes in PS II protein levels did not affect the proper functioning of PS II. The maximal PS II efficiency (F_v_/F_m_), the operating efficiency of PS II (ΦPSII), and photochemical quenching (qP) in *s2p2* mutants were similar to those in WT plants. The non-photochemical quenching (NPQ) and F_0_ parameters, however, significantly increased in both mutant lines. NPQ is a process related to heat dissipation, an excess of absorbed light energy (Maxwell and Johnson, 2000). The NPQ may increase when linear and cyclic electron flow is intensified causing the accumulation of protons in the thylakoid lumen. This results in thylakoid lumen acidification, which promotes the conversion of violaxanthin to zeaxanthin (Ruban et al., 2012; Yamamoto, 2016). This mechanism protects PS II from excess light (qE), and xanthophylls are carotenoids that play a critical part in such NPQ mechanism. The higher level of carotenoids measured in *s2p2* mutants may be one of the elements contributing to the observed increase in NPQ. The NPQ arises also in the absence of ΔpH when damaged reaction centers act as weak energy traps (qI) (Ruban et al., 2012). Therefore, the qI component may also play an important role in the observed enhancement of NPQ in the *s2p2* mutant lines. Although the observed increase in NPQ and F_0_ under non-stressful light conditions was not directly associated with a decrease in the F_v_/F_m_ value, the reduced content of PS II and LHC II proteins may be linked to an increased sensitivity of plants to photoinhibition. To investigate how variation in PsbA/D and PsbC abundance influences the PS II activity under photinhibitory conditions, a time-course experiment has been applied. Under high light conditions (1,000 μmol m^−2^ s^−1^), both *s2p2* mutant lines were characterized by a more rapid and deeper decrease of the F_v_/F_m_ parameter than WT plants. This decrease was clearly visible in both the presence and absence of lincomycin, which indicates the higher sensitivity of PS II particles from *s2p2* mutant lines to photodamage. Moreover, after restoring comfortable light conditions, the recovery rate observed in *s2p2* mutants was also significantly slower than in WT plants. The actual sensitivity to photoinhibition *in vivo* depends largely on the balance between PsbA inactivation and the regeneration process, which involves the incorporation of new PsbA molecules into the PSII complex (Vijayan and Browse, 2002). Replacing the damaged PsbA polypeptide with a new copy is a multistage process, which comprises the PS II–LHC II supercomplexes disassembled to monomeric forms and its migration to the stroma-exposed thylakoid membranes. The direct degradation of photodisturbed PsbA and substitution of a new copy required also partial disassembly of the PS II monomer (Järvi et al., 2015). Therefore, the balance between the rate of synthesis and the ability to degrade the photodamaged PsbA plays an important role, but these are not the only factors affecting the effectiveness of the recovery process. However, sensitivity to photoinhibition and recovery process are also dependent on other factors, such as temperature and the lipid composition of thylakoid membranes (Vijayan and Browse, 2002). Both investigated *s2p2* mutant lines have been characterized by the decreased levels of PsbA, PsbC, and PsbD polypeptides. The lower amount of available functional copies of these polypeptides, may be a direct cause for which the repair of photodamaged PS II complexes is a process that is significantly slower in *s2p2* mutants than in WT plants.

Summarizing, the presented results strongly indicate that S2P2 protease plays an important role in maintaining the proper functioning of PS II in both standard and elevated irradiance and is involved in plant response to high light stress. Therefore, S2P2 is another chloroplast intramembrane protease that, alongside other chloroplast intramembrane proteases such as EGY1, EGY2, and EGY3, plays a significant role in shaping and maintaining the proper stoichiometry of proteins building thylakoid photosynthetic complexes and in the plant’s response to adverse light conditions.

## Data availability statement

The original contributions presented in the study are included in the article/supplementary material. Further inquiries can be directed to the corresponding author.

## Author contributions

MC: Investigation, Methodology, Writing – original draft. MA: Data curation, Investigation, Methodology, Writing – original draft. RL: Conceptualization, Data curation, Formal analysis, Investigation, Methodology, Supervision, Writing – original draft, Writing – review & editing.
